# Robotic-assisted surgery in benign gynecology: single-center experience with 106 patients

**DOI:** 10.3389/fmed.2025.1677721

**Published:** 2025-11-27

**Authors:** Leïla Oujjat, Ralf Rothmund, Emin Aghayev, Christos Tsaousidis, Martin Mueller

**Affiliations:** 1 Faculty of Medicine, University of Bern, Bern, Switzerland; 2 Department of Gynecology and Obstetrics, Frauenzentrum Bern, Lindenhofspital AG, Bern, Switzerland; 3 Department of Research, Lindenhofgruppe AG, Bern, Switzerland; 4 Institute of Biochemistry and Molecular Medicine, University of Bern, Bern, Switzerland

**Keywords:** robotic-assisted surgery, Da Vinci robot, benign gynecological diseases, perioperative outcomes, learning curve

## Abstract

**Objective:**

Robotic-assisted surgery is increasingly used in gynecology, offering technological and ergonomic benefits that overcome the limitations of traditional laparoscopy. This retrospective study evaluates perioperative outcomes, learning curve, and feasibility of robotic-assisted surgery for benign gynecological indications using the Da Vinci Xi Surgical System (Intuitive Surgical) at the Department of Gynecology and Obstetrics at Frauenzentrum Bern, Lindenhofspital AG, Switzerland.

**Methods:**

Between August 2021 and November 2024, 106 consecutive patients underwent robotic-assisted surgery for various benign gynecological conditions. Clinical and perioperative outcomes were assessed. To evaluate the learning curve, the cohort was divided chronologically into two equal groups (first 53 and subsequent 53 cases). Differences between groups were analysed using multivariate logistic regression. The impact of potential predictors on total operation time was assessed using multivariate linear regression (significance level: *p* < 0.05). Predictors included: age, body mass index (BMI), uterine weight, parity, previous surgery, console time, operation time, conversion rate, blood transfusions, number of incisions, and patient group.

**Results:**

All cases were successfully completed robotically without conversion to laparotomy. No serious adverse events occurred. Logistic regression showed significant differences between the two groups in a 0.008-times heavier uterine weight (Confidence Intervals (CI) 0.003–0.014; *p* = 0.003), a 0.11-times higher BMI (CI 0.01–0.21; *p* = 0.032), a 0.06-times shorter console time (CI 0.01–0.10; *p* = 0.010), and a 1.91-times lower number of incisions (CI 0.43–3.39; *p* = 0.011) in the second group. Operation time did not differ significantly (*p* = 0.76). Linear regression showed that uterine weight and console time (both *p* < 0.001) were associated with operation time. Operation time increased by 7.6 (CI 5.2–9.9) minutes per 100 g of uterine weight, and by 7.8 (CI 5.5–9.8) minutes per 10 additional minutes of console time.

**Conclusion:**

Robotic-assisted surgery is a safe and feasible alternative to conventional laparoscopy in benign gynecology. The learning curve was demonstrated by reduced console time and fewer incisions in later cases, despite higher BMI and uterine weight. Further studies should assess patient benefits and cost-effectiveness compared to conventional laparoscopy.

## 1 Introduction

Robotic-assisted surgery is a further approach to minimally invasive surgery and has gained increasing relevance in gynecology over the past two decades (1, 2). Initially introduced in neurosurgery, urology, and cardiac surgery, its application in gynecologic procedures such as hysterectomy, myomectomy, sacrocolpopexy, complex endometriosis surgery, and treatment of malignancies, especially endometrial cancer, has expanded rapidly since the early 2000s (2–5). Among these, robotic-assisted laparoscopic hysterectomy is the most commonly performed robotic procedure in gynecology. Robotic-assisted surgery preserves the advantages of minimally invasive approaches while offering technological and ergonomic benefits that address limitations of traditional laparoscopy, including two-dimensional imaging, suboptimal surgeon ergonomics, hand tremors, and the restricted mobility of rigid laparoscopic instruments (3, 6, 7). The most widely used surgical robot is the Da Vinci Surgical System, approved by the US Food and Drug Administration for gynecological surgery in 2005 (7, 8).

Gynecological disorders encompass a wide range of benign and malignant diseases affecting the female reproductive organs. Among these, conditions such as uterine fibroids, endometriosis, adenomyosis, and precancerous lesions of the cervix are of particular clinical relevance due to their high prevalence and potential impact on reproductive health and quality of life. The development and integration of robotic-assisted surgical techniques in gynecology have considerably advanced the management of these disorders. Uterine fibroids, or leiomyomas, are the most common benign tumors in women of reproductive age (9). Management strategies are tailored to symptom severity and include surgical options such as myomectomy or hysterectomy (10). Another frequent diagnosis is endometriosis, which is a chronic inflammatory disease in which ectopic lesions are most commonly found on the ovaries, pelvic peritoneum, rectovaginal septum, bowel, bladder, and in the pouch of Douglas (11, 12). Deep infiltrating endometriosis (DIE), defined as endometrial-like tissue extending on or under the peritoneal surface, may affect adjacent organs and disrupt normal pelvic anatomy by inducing fibrosis, adhesions, and changes in vascularization, thereby increasing surgical complexity. It often necessitates meticulous dissection near critical pelvic structures, extensive adhesiolysis, and adaptation to distorted anatomical planes. According to a recent review by Ferrari F et al. (13), the robotic platform’s features - three-dimensional visualization, wristed instrumentation, tremor filtration, and improved ergonomics - may enhance precision and safety in these challenging cases, although evidence remains limited and the risk–benefit balance continues to be evaluated. Moreover, deep endometriosis can prolong console time, influence docking strategies, and elevate complication risk, emphasizing the need to assess robotic performance not only in routine benign indications but also in complex pathologies such as endometriosis (13). Adenomyosis, although histologically distinct from endometriosis, often coexists with it and shares overlapping clinical features. Both endometriosis and adenomyosis are challenging due to their overlap with uterine fibroids, and up to one third of the patients may be asymptomatic (14, 15). Surgical management may involve uterine-sparing techniques such as laparoscopic or robotic-assisted excision, endometrial ablation, uterine artery embolization or, in severe cases, hysterectomy (16, 17). Finally, precancerous lesions - particularly cervical intraepithelial neoplasia (CIN) and endometrial hyperplasia - represent another common indication for surgery.

In this retrospective study, we evaluated our experience using the Da Vinci Xi Surgical System (Intuitive Surgical) for various benign indications in the first 106 consecutive surgeries. We analysed the clinical characteristics, perioperative outcomes, learning curve, and feasibility of robotic-assisted surgery in the cohort treated between August 2021 and November 2024 at the Department of Gynecology and Obstetrics at Frauenzentrum Bern, Lindenhofspital AG, Switzerland.

## 2 Materials and methods

### 2.1 Retrospective data analysis

We identified 106 consecutive patients who underwent various robotic-assisted surgeries for benign gynecological diseases between August 2021 and November 2024 at the Department of Gynecology and Obstetrics at Frauenzentrum Bern, Lindenhofspital AG, Switzerland, with the aim of analysing clinical outcomes and the surgeon’s learning curve. Notably, the Da Vinci Xi Surgical System (Intuitive Surgical) was introduced in our institution in August 2021, and all patients with benign gynecological diseases, who were eligible for a minimally invasive procedure, were considered for the robotic-assisted surgery. Eligible patients were preoperatively informed about the advantages, disadvantages, and limitations of robotic-assisted surgery and provided written informed consent in accordance with institutional ethical approval. Final patient selection was based on a shared decision-making process between the surgeon and the patient, which may have introduced a potential selection bias. We used the Da Vinci Xi Surgical System in all the cases and all procedures were performed by the same surgeon (T.C.), an experienced laparoscopic surgeon who had completed a certified training course in robotic-assisted gynecologic surgery. All patient records were retrospectively reviewed to identify clinical characteristics and perioperative outcomes. The clinical characteristics include age, body mass index (BMI), parity, previous abdominal or pelvic surgery, diagnosis, indication and type of surgery. The perioperative outcomes reported include operation time (measured in minutes from skin incision to wound closure, including the times for both docking and undocking the robotic arms), console time (measured in minutes during which the operating surgeon used the robotic console), number of incisions, uterine weight, length of hospital stay, adverse events, blood transfusions, and conversion to laparotomy. Additionally, we assessed the learning curve by splitting the cohort chronologically into two equally large groups of 53 cases each.

### 2.2 Statistical methods

We present the results as the mean ± standard deviation (mean ± SD). In order to assess the learning curve, we divided the cohort chronologically into two equally large groups: first 53 and subsequent 53 cases.

Missing data were assessed for mechanism and extent. There were missing values for the following parameters: Body mass index (BMI) (2/106, 1.9%), uterine weight (4/106, 3.8%), console time (5/106, 4.7%), and parity (11/106, 10.3%). We first tabulated missingness by variable and subgroup and applied Little’s test to evaluate the Missing Completely At Random (MCAR) assumption (mcartest statement in Stata). All tests confirmed the null-hypothesis of data MCAR. Subsequently, to improve precision and account for uncertainty due to missing values, we conducted a linear interpolation on patient age (ipolate statement in Stata; for BMI, console time and parity) or BMI (for uterine weight) to estimate the missing values.

We analysed the influence of potential predictors on operation time, being a continuous variable, using multivariate linear regression. We included all available observed factors as potential predictors in the regression: age, BMI, uterine weight, parity, previous surgery, console time, operation time, conversion rate, blood transfusions, number of incisions, and patient group. Subsequently, multivariate logistic regression analysis was carried out to evaluate differences between the two patient groups, being a binary variable.

The analyses were carried out in Stata (version 18.0). The significance level was set at *p* < 0.05.

### 2.3 Operating room setup and surgical procedure

At least two surgeons were present to perform all the procedures. The main surgeon (T.C.) was stationed at the robotic console remote from the patient but within the same operating room. The assisting surgeon was stationed at the patient’s side. All cases were performed under general anesthesia. The patient was placed in a dorsal lithotomy position. Vaginal disinfection treatment and disinfection of the surgical site were carried out. This was followed by the insertion of a transurethral indwelling catheter and a uterine manipulator, if the patient had a uterus. Then followed the sterile draping of the patient. An incision of the umbilical fossa was made with the 11 mm scalpel. After obtaining adequate pneumoperitoneum by inserting a Veress needle at the umbilicus, two or three additional periumbilical incisions were made for the insertion of the trocars. Once all desired ports were in place, the patient was placed in a steep Trendelenburg position. The robotic arms were then docked onto the trocars. Upon abdominal entry with the high-definition camera which provides a three-dimensional imaging, visualization of the abdominal cavity was done. Then followed the respective surgical procedure. The bedside surgeon was responsible for the exchanges of the EndoWrist^®^ instruments and the management of any accessory port activity such as providing suction or irrigation. After all surgical procedures, adhesion prophylaxis was carried out by rinsing the abdominal cavity with saline solution. The robotic arms were undocked, and the trocars removed. The incisions were closed using subcuticular 3.0 Monocryl sutures (18–21).

## 3 Results

We identified a total of 106 patients who underwent various robotic-assisted surgeries for benign gynecological diseases in the above given period.

### 3.1 Patient characteristics and indications

The patient’s clinical characteristics are displayed in Table 1. The surgical indications and diagnoses are summarized in Supplementary Table 1.

**TABLE 1:** In this table, patient clinical characteristics are compared between the first half (Group 1) and the second half (Group 2) of the study cohort, with results further stratified by type of surgical procedure.

Procedures	All surgeries (*n* = 106)	HE + SE (*n* = 74)		HE + AE (*n* = 26)		HE + AE + UPS (*n* = 2)		AE (*n* = 3)		Endometriosis (*n* = 1)	
		Group 1	Group 2	Group 1	Group 2	Group 1	Group 2	Group 1	Group 2	Group 1	Group 2
Number (n)	106	41	33	12	14	0	2	0	3	0	1
Age (mean ± SD), years	50 ± 11	45 ± 7	44 ± 6	63 ± 10	62 ± 8	–	71 ± 18	–	64 ± 10	–	44
BMI (mean ± SD), kg/m2	26.7 ± 5.2	26 ± 4	27 ± 4*	26 ± 5	29 ± 7*	–	32 ± 9	-	27 ± 11	–	19
Parity (%)	74.5	68.3	84.8	75	71.4	–	100	–	66.7	–	0
Previous abdominal or pelvic surgery (%)	57.5	65.8	51.5	41.7	50	–	100	–	66.7	–	100

The table presents the distribution of demographic and clinical variables, including age, body mass index, parity and previous abdominal or pelvic surgery. Data are presented as mean ± standard deviation. Statistically significant differences between groups are indicated by *p < 0.05. *n*, number; SD, standard deviation; BMI, body mass index; HE, hysterectomy; SE, salpingectomy; AE, adnexectomy; UPS, unilateral pectineal suspension.

A total of 106 patients with a mean age (±SD) of 50 ± 11 years and body mass index of 26.7 ± 5.2 kg/m^2^ were treated for benign gynecological diseases, such as uterine fibroids [46% (49/106)], adenomyosis [43% (46/106)], cervical dysplasia [17% (18/106)], endometrial hyperplasia [8% (9/106)], ovarian cyst [6.6% (7/106)], endometriosis [6.6% (7/106)], uterine polyps [5.6% (6/106)], BRCA mutation [2% (2/106)], descensus of the uterus [2% (2/106)], bicornate uterus [2% (2/106)] and uterine lipoleiomyoma [1% (1/106)]. The indications for the surgeries included bleeding disorders (such as hypermenorrhea, menometrorrhagia and postmenopausal bleeding) not responding to medical therapy, pain (such as dysmenorrhea and chronic pelvic pain), pelvic organ prolapse, and risk reduction. Several patients had more than one diagnosis and most patients had more than one indication for the surgery.

66% (70/106) of the patients had at least one parity, of which 34% (24/70) had caesarean section. 57.5% (61/106) had previous abdominal or pelvic surgeries including caesarean section [39% (24/61)], appendectomy [28% (17/61)], hernia surgery [21% (13/61)], laparoscopic endometriosis surgery [11% (7/61)], laparoscopic sterilization [10% (6/61)], adnexectomy [8% (5/61)], bariatric surgery [8% (5/61)], cholecystectomy [6.5% (4/61)], laparoscopic ovarian cyst removal [3% (2/61)], myoma resection [3% (2/61)], transabdominal disc hernia surgery [1.6% (1/61)], laparoscopic adhesiolysis [1.6% (1/61)], hysterectomy [1.6% (1/61)], laparoscopic diverticulum surgery [1.6% (1/61)] and lipoma resection [1.6% (1/61)].

### 3.2 Procedure outcomes

#### 3.2.1 Surgical procedures

In our cohort the most common procedure was hysterectomy. We performed 102 cases of hysterectomy for benign indications of which 100 were total and two supracervical. We performed additional procedures such as salpingectomy [72.5% (74/102)], adnexectomy [25% (26/102)], and adnexectomy + unilateral pectineal suspension [2% (2/102)]. Other surgical procedures for benign indications (without hysterectomy) were three cases of adnexectomy, two of them bilaterally and one unilaterally with additional myoma resection, and one case of removal of endometriosis cyst. We performed adhesiolysis in 21% (22/106) of the cases.

The mean values (mean ± SD) of the perioperative outcomes are summarized in Table 2.

**TABLE 2:** In this table, perioperative outcomes are compared between the first half (Group 1) and the second half (Group 2) of the study cohort, with results further stratified by type of surgical procedure.

Procedures	All surgeries (*n* = 106)	HE + SE (*n* = 74)		HE + AE (*n* = 26)		HE + AE + UPS (*n* = 2)		AE (*n* = 3)		Endometriosis (*n* = 1)	
		Group 1	Group 2	Group 1	Group 2	Group 1	Group 2	Group 1	Group 2	Group 1	Group 2
Operation time (mean ± SD, CI*), min	8833, 82–94	93 ± 37	86 ± 32	90 ± 30	79 ± 24	–	99 ± 6	–	70 ± 42	–	75
Console time (mean ± SD, CI*), min	52 ± 23, 48–57	60 ± 29	49 ± 20*	55 ± 18	42 ± 13*	–	62 ± 4	–	28 ± 5	–	33
Number of incisions (mean ± SD, CI*)	3.8 ± 0.4, 3.75–3.9	4 ± 0	4 ± 0*	4 ± 0	4 ± 0*	–	4	–	3 ± 1	–	4
Uterine weight (mean ± SD, CI*), g	185 181, 150–219	178 ± 162	248244*	95 + 64	138 ± 92*	–	80 ± 16	–	270 ± 92	–	71
Length of hospital stay	2.2 ± 0.4, 2.1–2.2	2 ± 0	2 ± 0	2 ± 0	2 ± 0	–	2 ± 1	–	2 ± 0	–	2
Intraoperative complications (n)	0	0	0	0	0	–	0	–	0	–	0
Postoperative complications (n)	5	1	4	0	0	–	0	–	0	–	0
Blood transfusions (n)	0	0	0	0	0	–	0	–	0	–	0
Conversion rate (n)	0	0	0	0	0	–	0	–	0	–	0

The table presents key perioperative variables, such as operation and console time. Data are presented as mean ± standard deviation. Statistically significant differences between groups are indicated by *p < 0.05. *n*, number; SD, standard deviation; CI, 95% confidence intervals; HE, hysterectomy; SE, salpingectomy; AE, adnexectomy; UPS, unilateral pectineal suspension.

#### 3.2.2 Length of the procedures

We evaluated both the operation time, measured in minutes from skin incision to wound closure, including the times for both docking and undocking the robotic arms, and the console time, measured in minutes during which the operating surgeon used the robotic console.

##### 3.2.2.1 Operation time

The mean operation time (±SD) for all types of surgery was 88 ± 33 min. Regarding the different types of surgery, the mean operation time was 99 ± 6 min for hysterectomy + adnexectomy + unilateral pectineal suspension, 90 ± 35 min for hysterectomy + salpingectomy, 84 ± 27 min for hysterectomy + adnexectomy, 75 min for the removal of an endometriosis cyst, and 70 ± 42 min for adnexectomy. The procedure with the longest operation time (226 min) was a hysterectomy with salpingectomy in a patient with large fibroids (uterine weight 446 g) requiring intraabdominal morcellation of the uterus. This was the first case performed by the surgeon with the Da Vinci robot since it was introduced in our institution.

##### 3.2.2.2 Console time

The mean console time (±SD) for all types of surgery was 52 ± 23 min. Regarding the different types of surgery, the mean console time was 62 ± 4 min for hysterectomy + adnexectomy + unilateral pectineal suspension, 55 ± 26 min for hysterectomy + salpingectomy, 48 ± 16 min for hysterectomy + adnexectomy, 33 min for the removal of an endometriosis cyst, and 28 ± 15 min for adnexectomy.

The procedure with the longest console time (142 min) was a hysterectomy with salpingectomy in a patient with large fibroids (uterine weight 783 g) requiring vaginal morcellation of the uterus.

#### 3.2.3 Number of incisions

The mean number of incisions (±SD) was 3.8 ± 0.4, including the incision in the belly button.

#### 3.2.4 Uterine weight

The mean uterine weight (±SD) was 185 ± 181 g. The heaviest uteri occurred in patients with uterine fibroids, with the heaviest weighing 1166 g.

#### 3.2.5 Length of hospital stay

The mean hospital stay (±SD) was 2.2 ± 0.4 nights after the surgery.

All patients were discharged from hospital in good conditions.

#### 3.2.6 Adverse events

We report a complication rate of 4.7%. No intraoperative complications occurred in any patients. The postoperative course was unremarkable for the entire cohort except in five patients.

According to the published literature, following adverse events could be expected in robotic-assisted gynecological surgery: Intraoperative complications such as bladder injuries, bowel injuries, vessel injuries and arm compression, and postoperative complications such as vaginal cuff hematoma, vaginal cuff dehiscence, port-site hernia, periumbilical hematoma, surgical site infections, febrile events, pulmonary edema, ureterovaginal fistula, and peritonitis (21–23). In our cohort, one patient presented bleeding from the vaginal cuff under acetylsalicylic acid (ASA) in postoperative week 4, requiring a surgical revision of the suture. Two patients developed an infected vaginal hematoma, requiring a laparoscopic drainage and antibiotic therapy, respectively. One patient presented on the 27th postoperative day with a pulmonary embolism, whereupon intensive anticoagulation therapy was started immediately. Another patient developed a deep vein thrombosis in the leg with known primary varicosis. This was treated with anticoagulation and compression stockings. These adverse events were diagnosed either during hospital stay or after patient discharge. All cases were successfully completed with robotic assistance with no need for a conversion to laparotomy. No blood transfusions were required.

#### 3.2.7 Learning curve

We compared the patient’s clinical characteristics and perioperative outcomes of our chronologically first 53 cases (Group 1) to subsequent 53 cases (Group 2). Significant differences were found in body mass index, uterine weight, console time and number of incisions (Tables 1, 2). Body mass index increased by 5% (26 vs. 27.4) and uterine weight by 31.5% (159 vs. 210 g) in Group 2. Despite this, console time decreased by 28% (58.9 vs. 45.8 min). In terms of the number of incisions, it decreased by 5% (3.9 vs. 3.7).

#### 3.2.8 Multivariate regression analyses

**FIGURE 1:**
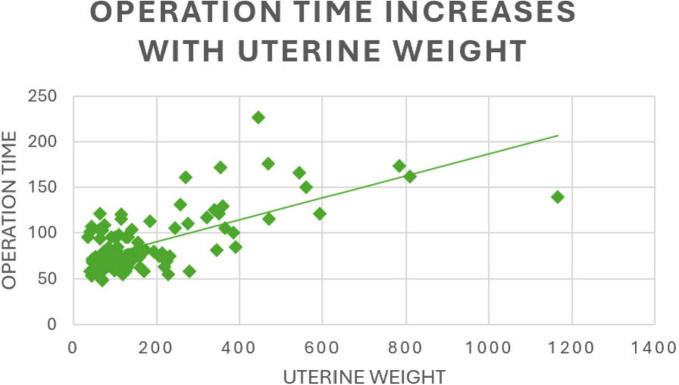
This figure presents a linear regression analysis of the relationship between uterine weight and operation time. The analysis shows that operation time increases with greater uterine weight.

Linear regression analysis confirmed that uterine weight and console time were significantly associated with operation time (both *p* < 0.001). Operation time increased by 7.6 min (Confidence Intervals [CI] 5.2–9.9 min) per 100 g of uterine weight (Figure 1), and by 7.8 min (CI 5.5–9.8 min) per 10 additional minutes of console time. As for the remaining potential predictors, no significant association with operation time is observed in the multivariate analysis (*p* ≥ 0.06; Table 3).

**TABLE 3:** In this table, the results from the multivariate linear and logistic regression models are presented for each parameter.

Parameter	*p*-value		Lower Cl		Upper CI		Estimate	OR
	Linear model	Logistic model	Linear model	Logistic model	Linear model	Logistic model	Linear model	Logistic model
Parity (per child)	0.42	0.37	−1.9	−0.22	4.6	0.59	1.4	0.18
Uterine weight (per 100 g)	0.000	0.003	5.2	0.003	9.9	0.014	7.6	0.008
Console time	0.000	0.010	5.5	−0.10	9.8	−0.01	7.8	−0.06
Body mass index	0.84	0.032	−0.8	0.01	0.6	0.21	−0.1	0.11
Number of incisions	0.48	0.011	−6.5	−3.39	13.7	−0.43	3.6	−1.91
Age (per 10 years)	0.20	0.29	−1.2	−0.02	5.5	0.07	2.2	0.02
Previous surgeries (per surgery)	0.06	0.56	−0.2	−0.27	5.6	0.51	2.7	0.12
Group	0.65	0.76	−39	−0.04	55.3	0.03	−1.9	−0.01

Estimates, odds ratios (OR), and 95% confidence intervals (CI) are shown to summarize the associations with the outcomes of interest. Parameters include parity, uterine weight, console time, body mass index, number of incisions, age, previous surgeries, and study group. Statistically significant associations can be identified by the corresponding *p*-values and confidence intervals.

Logistic regression analysis revealed no significant differences between the two groups in patient age, parity, the number of previous surgeries, and operation time (*p* ≥ 0.29; Table 3). Significant differences were observed in uterine weight, BMI, console time, and in number of incisions. The second group had a 0.008-times heavier uterine weight (CI 0.003–0.014; *p* = 0.003), a 0.11-time higher BMI (CI 0.01–0.21; *p* = 0.032), a 0.06-times shorter console time (CI 0.01–0.10; *p* = 0.010), and a 1.91-times lower number of incisions (CI 0.43–3.39; *p* = 0.011). The operation time was not significantly different between the two groups (*p* = 0.76).

## 4 Discussion

Robotic-assisted surgery combines the benefits of minimally invasive surgery with advanced technological and ergonomic features. The clinical benefit over conventional laparoscopy remains under debate, especially in terms of outcomes such as operation time, blood loss, complication rates, and recovery.

We successfully completed all 106 cases without a conversion to laparotomy. No serious adverse events were detected. Importantly, the mean operation and console times were shorter than those reported in the literature despite the increasing uterine weight and body mass index (see Table 2) (24–26). We achieved these shorter times for several reasons. First, all surgeries were performed by a single experienced surgeon and a highly skilled team. Second, we perform a large number of cases per year, which positively impacts the learning curve and overall progress (24). However, direct comparison with other studies is challenging for several reasons. Study design, case selection, and technological platform differ substantially across published series. In particular, studies using the Da Vinci SP System often report longer operation and console times compared with those using the Da Vinci Xi System, as used in our department, due to the SP system’s single-port setup, restricted triangulation, and longer docking time (25, 27). Furthermore, reported outcomes may vary depending on the degree of surgeon experience, the number of operating surgeons per study, and institutional factors such as team consistency and standardization of workflow. In addition, total operation time is influenced by multiple variables beyond surgical expertise, such as surgical technique and approach, as well as patient-related factors including obesity, age, increased uterine weight, and extensive adhesions (28).

The surgeon’s learning curve is summarized in Tables 1, 2. Although total operation time remained nearly identical between Group 1 (chronologically first 53 cases) and Group 2 (subsequent 53 cases), this finding must be interpreted with caution, as increasing case complexity in the later cohort likely masked true improvements in surgical efficiency. Specifically, Group 2 included cases with significantly heavier mean uterine weight (+31.5%) and higher BMI (+5%) - both well-established predictors of prolonged operative duration and increased technical difficulty. In addition, the second group contained a higher proportion of complex procedures requiring additional steps such as adhesiolysis and uterine morcellation, which further prolonged total operation time. Our multivariate linear regression confirmed that uterine weight and console time were independent predictors of total operation time (both *p* < 0.001). Operation time increased by 7.6 min per 100 g of uterine weight and by 7.8 min per additional 10 min of console time, highlighting that procedure- and patient-related factors significantly influence total operation time beyond the learning curve itself. When analysing a subgroup of cases with comparable uterine weights (approximately 400 g), we observed noticeably shorter total operation times in later cases compared to earlier ones. This suggests that surgical performance and workflow efficiency indeed improved with experience, but these improvements were partially concealed in the overall analysis due to the progressive inclusion of more complex cases.

This confounding effect represents an inherent limitation of retrospective learning-curve studies: as surgeons gain confidence, they tend to select increasingly challenging cases, which can obscure measurable improvements in total operation time. Interestingly, when isolating procedure-specific variables, the console time decreased significantly by 28% in the second group, clearly indicating improved efficiency in the robotic-assisted components of the surgery. This finding supports the concept that increasing familiarity with the robotic system – particularly in docking, instrument handling, and console-based dissection—leads to measurable procedural improvements over time. Importantly, in complex cases the surgeon left the console to perform vaginal or abdominal morcellation of large uteri, which added to total operation time but not to console time. This distinction highlights that console time may be a more reliable indicator for assessing the learning curve in robotic-assisted surgery than total operation time, especially when comparing cohorts with differing case complexity.

Our findings align with several previously published learning curve analyses (19, 22, 26, 29–34). The study by El Hachem et al. (19) reported a 28% and 39% reduction of the operation and console time in Cohort 2 compared to Cohort 1 (113 vs. 81 and 59 vs. 36). Similarly, Favre et al. (22) observed a 20% decrease in operation time (156.8 vs. 125.8) between the two cohorts, while Jayakumaran et al. (33) noted a 32% reduction of the console time in Cohort 2 compared to Cohort 1 (59.3 vs. 40.4). Compared to these studies, our observed 10% decrease in total operation time appears modest, but this is plausibly explained by the increased complexity and heavier uteri in our later cases. The subgroup analysis further supports this interpretation, demonstrating shorter operation times in later cases of similar complexity, confirming that true procedural improvement occurred despite more demanding case selection.

We report a low complication rate of 4.7% compared to rates observed in recent studies.

The retrospective study by Favre et al. (22) reported a complication rate of 12.3% including intra- and postoperative events such as bladder and bowel injuries, vaginal cuff dehiscences, hematomas, and surgical site infections. The prospective cohort study by Marengo et al. (21) reported a complication rate of 12% including transitory ischemic attack, hernia, periumbilical hematoma, and vaginal cuff hematoma. The study by Yim et al. (35) reported a complication rate of 18.8% including bladder and vessel injuries, vaginal cuff bleeding and infection, febrile events, and uretrovaginal fistula. However, it should be noted that the present study is retrospective, which may introduce bias. We acknowledge this limitation.

Direct comparison between robotic-assisted and conventional laparoscopic surgery is of interest. The study by Sarlos et al. (1) reported no significant differences regarding perioperative outcomes such as complication rate, conversion to laparotomy, intraoperative bleeding and hospital stay. However, they demonstrated that operation times were significantly longer and operating room costs higher in the robotic group. As conventional laparoscopy was performed more frequently in their institution, they suggested that greater experience with the robotic platform could reduce operative duration. A study by Lee et al. (25) observed comparable complication rates, hospital stay, and pre- and postoperative hemoglobin levels between robotic and laparoscopic procedures, but reported longer operation times and higher postoperative C-reactive protein levels in robotic cases, potentially due to a larger umbilical incision and associated inflammatory response. Beyond these perioperative outcomes, robotic-assisted surgery generally entails higher direct costs from specialized instruments and system maintenance. Nonetheless, potential benefits have been described, including reduced postoperative pain, lower analgesic requirements, faster functional recovery, and comparable or slightly shorter hospital stay compared to conventional laparoscopy. Improved surgical proficiency reduces operation and anesthesia times, minimizes perioperative stress, and accelerates patient mobilization and recovery, collectively contributing to shorter hospitalization (24, 26, 27). Since our study did not directly compare the two approaches, we hypothesize that similar trends would apply in our cohort.

The present study has several limitations. First, since this report includes various surgical procedures, the description of a learning curve may be quite subjective. It was limited by the heterogeneity of the performed procedures and the specific preferences of the surgeon may have influenced the characteristics of the patients eligible for robotic-assisted surgery, which may lead to a selection bias.

An analysis of the learning curve stratified by surgical type (e.g., hysterectomy vs. adnexectomy) was not conducted due to the limited number of cases in each subgroup. Such an analysis would likely be influenced by random variation rather than reflecting true learning effects. Future studies with larger sample sizes should aim to explore this aspect to determine whether learning curves differ between specific surgical procedures. Second, due to the retrospective design we may not have covered all late postoperative complications that occurred. However, the study was strengthened by the fact that the patient population represents consecutive cases all performed by one experienced laparoscopic surgeon.

Our results support the concept that procedural performance improves with repetition and familiarity with the robotic platform. Surgeons not only become more adept at docking and manipulating the robotic instruments but also develop better workflow efficiency with their operating teams. Nevertheless, a performance plateau may occur as the complexity of selected cases increases—particularly in patients with conditions that would previously have been managed via open or conventional laparoscopic approaches.

## 5 Conclusion

The results confirm that robotic-assisted surgery is a safe and feasible approach for patients with benign gynecological diseases. We confirmed the presence of a learning curve by a decreased console time and the need of a lower number of incisions despite the presence of heavier uteri and a higher body mass index in the second group. Well-designed studies comparing robotic-assisted with conventional laparoscopic surgery are needed to characterize the patient benefits and the cost-effectiveness of robotic-assisted procedures.

## Data Availability

The original contributions presented in this study are included in this article/Supplementary material, further inquiries can be directed to the corresponding author.

## Supplementary material

Supplementary Table 1 In this supplementary table, the diagnoses and indications for the surgical procedures are summarized. The number of patients for each diagnosis is presented.
