# Supplementary figures and images for: Robotic-assisted surgery in benign gynecology: single-center experience with 106 patients
Source: Front Med (Lausanne). 2025 Nov 27;12:1677721. doi: 10.3389/fmed.2025.1677721 (PMC12695731; doi:10.3389/fmed.2025.1677721)

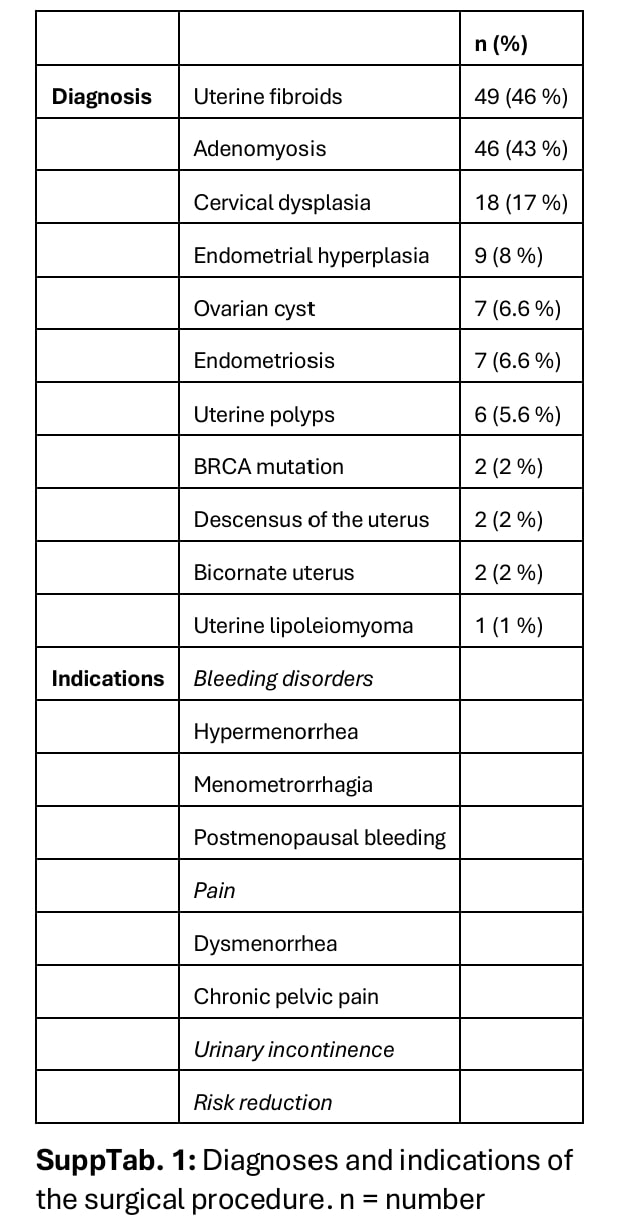

Supplement: Supplementary Table 1 — In this supplementary table, the diagnoses and indications for the surgical procedures are summarized. The number of patients for each diagnosis is presented. [file Image_1.jpeg]
